# Supplementary figures and images for: PerTurboID, a targeted in situ method reveals the impact of kinase deletion on its local protein environment in the cytoadhesion complex of malaria-causing parasites
Source: eLife. 2023 Sep 22;12:e86367. doi: 10.7554/eLife.86367 (PMC10564455; doi:10.7554/eLife.86367)

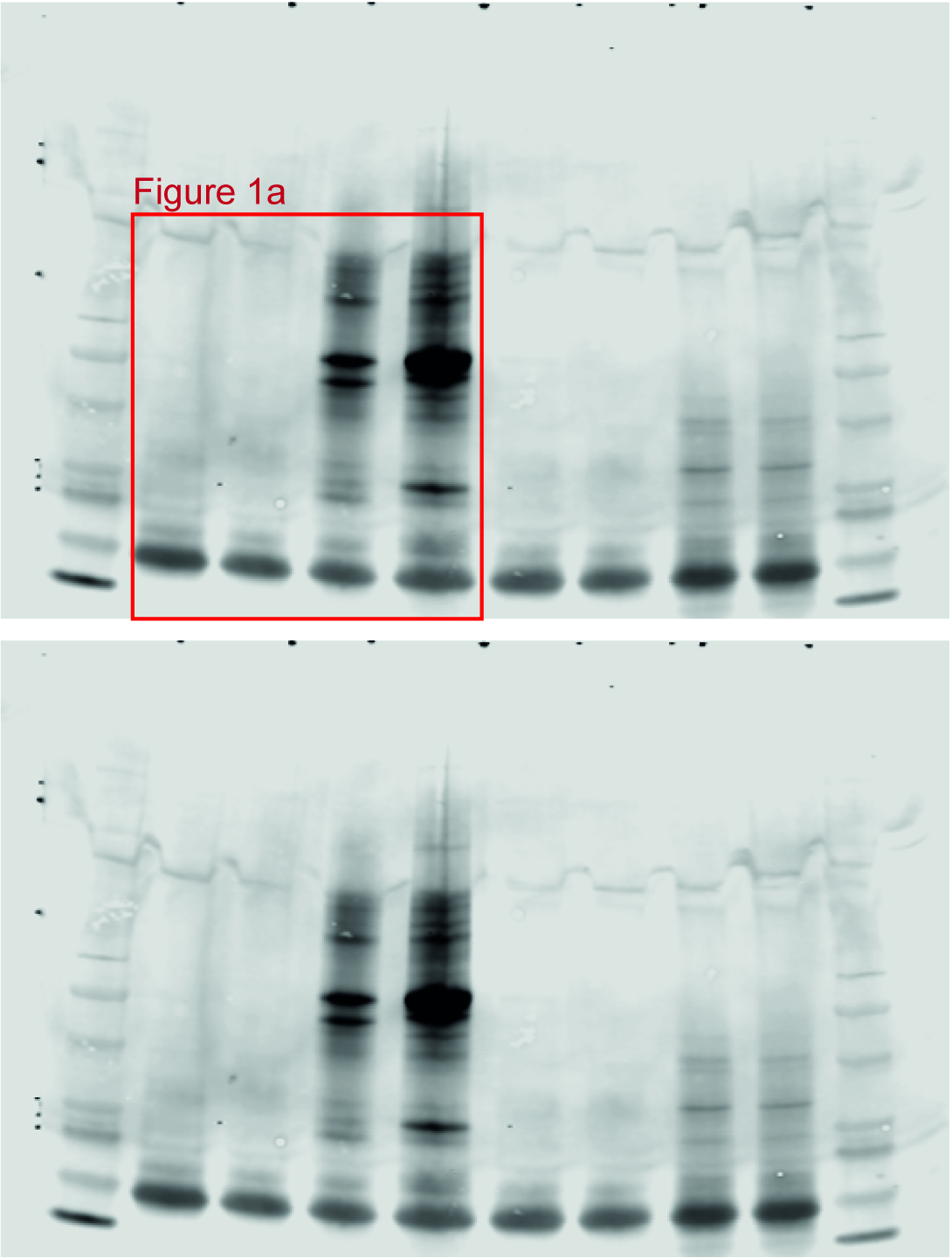

Supplement: Figure 1—source data 1. [file elife-86367-fig1-data1.zip › Figure 1-source data 1.tif]

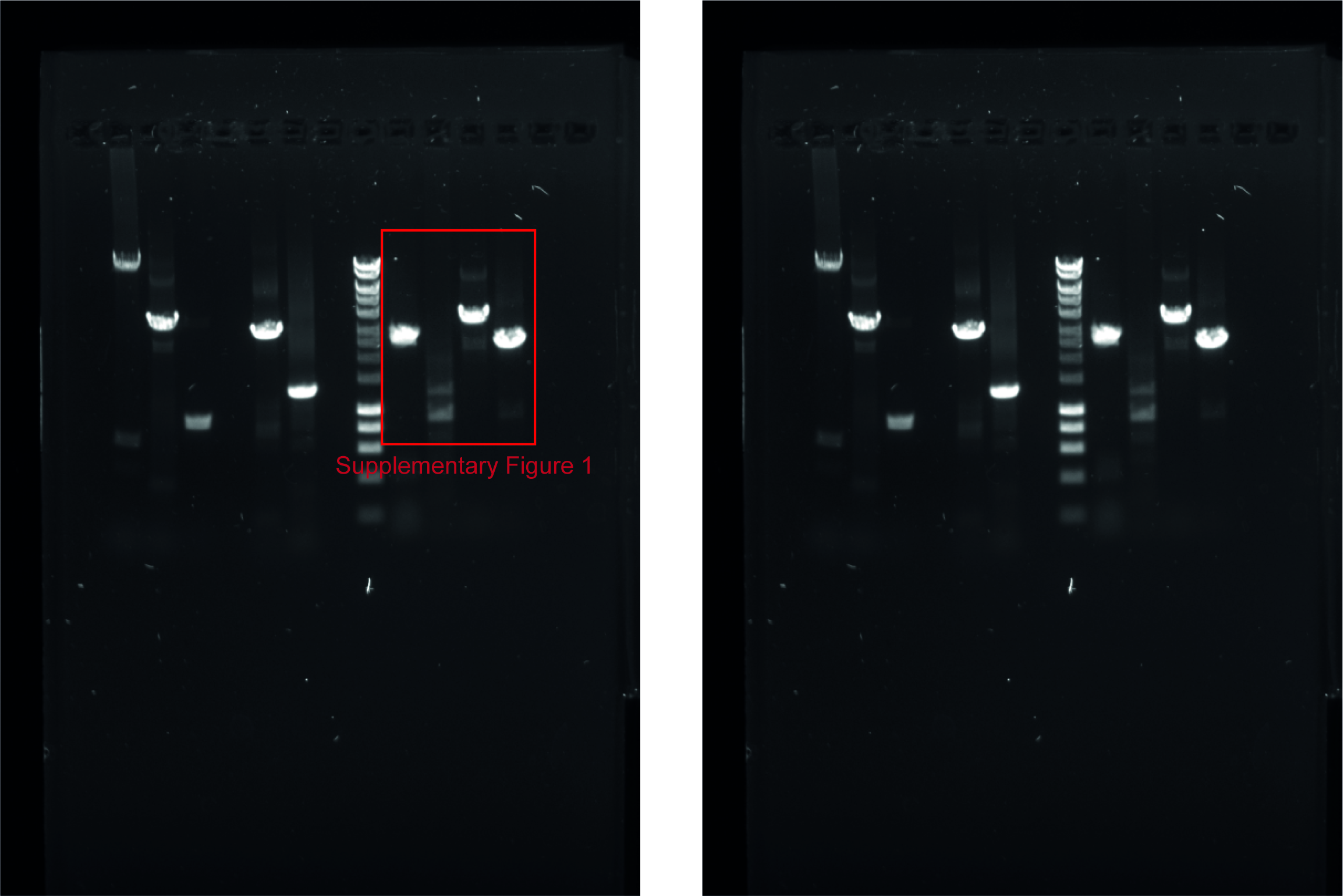

Supplement: Figure 1—figure supplement 1—source data 1. [file elife-86367-fig1-figsupp1-data1.zip › Figure 1- figure supplement 1 - source data 1.tif]

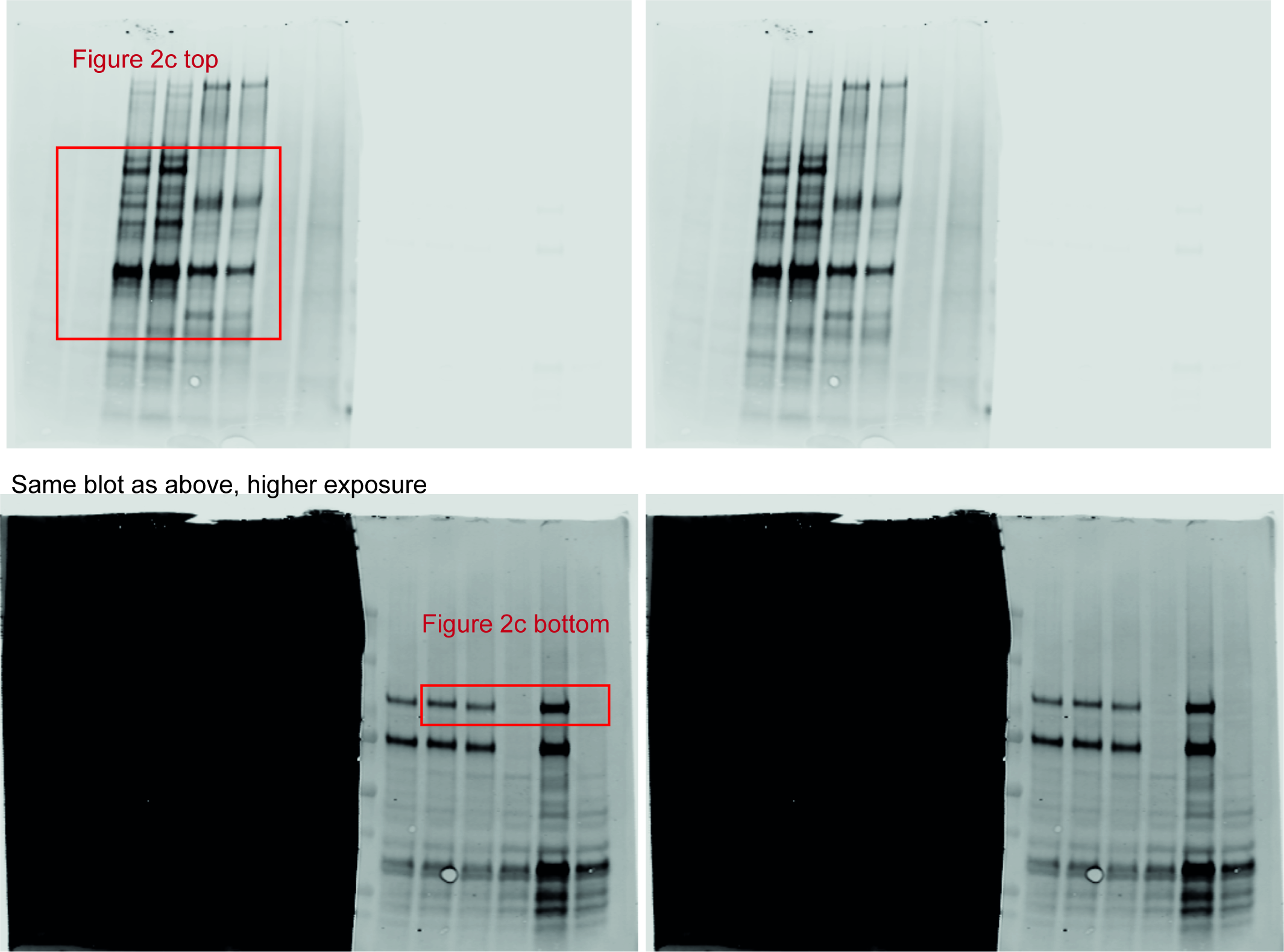

Supplement: Figure 2—source data 1. [file elife-86367-fig2-data1.zip › Figure 2- source data -1 .tif]

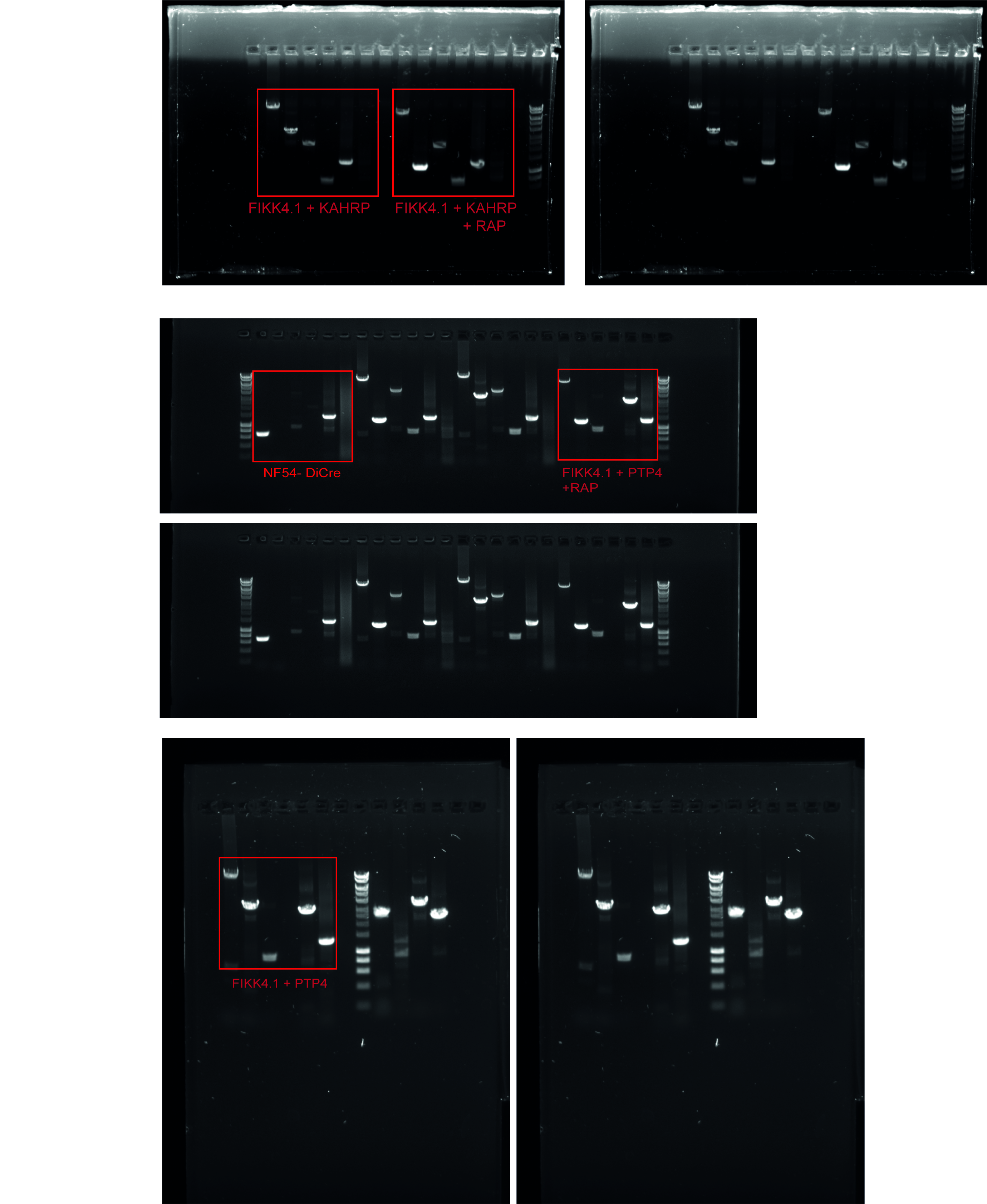

Supplement: Figure 2—figure supplement 1—source data 1. [file elife-86367-fig2-figsupp1-data1.zip › Figure 2- figure supplement 1 - source data 1.tif]

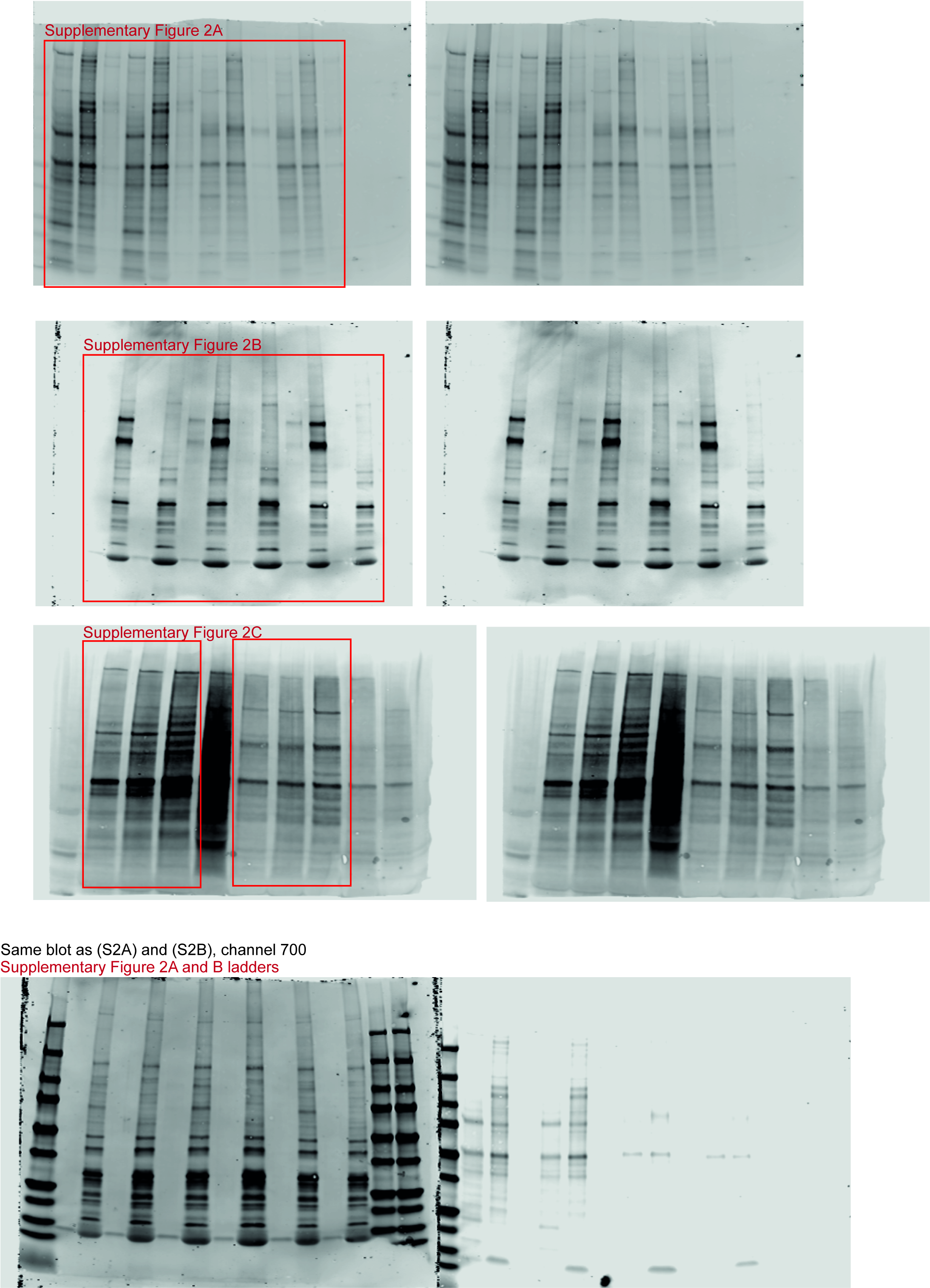

Supplement: Figure 2—figure supplement 3—source data 1. [file elife-86367-fig2-figsupp3-data1.zip › Figure 2- figure supplement 3 - source data 1.tif]
